# Effect of weaning strategies on biosynthesis of oxylipids in Holstein dairy calves

**DOI:** 10.3168/jdsc.2024-0600

**Published:** 2024-09-18

**Authors:** A.R. Wolfe, B.C. Agustinho, M. Mahdavi-Yekta, G.A. Contreras, D.E. Konetchy, A.H. Laarman, P. Rezamand

**Affiliations:** 1 Department of Animal, Veterinary and Food Sciences, University of Idaho, Moscow, ID 83844; 2 Department of Large Animal Clinical Science, Michigan State University, East Lansing, MI 48824; 3 Department of Agricultural, Food and Nutritional Science, University of Alberta, Edmonton, AB, Canada T6G 2P5

## Abstract

The aim of this study was to determine the effect of the weaning calves at 2 ages (early vs. late) and 2 weaning paces (abrupt over 3 d vs. gradual over 14 d) on plasma oxylipids. Seventy-one dairy calves (38.8 ± 4.4 kg, BW ± SD), blocked by sex and BW at birth, were randomly assigned in a 2 × 2 factorial arrangement of treatments. The factors were weaning age (early: 6–7 wk vs. late: 8–9 wk) and weaning pace (abrupt: 3 weaning steps over 2 d vs. gradual: 7 weaning steps over 14 d), generating 4 treatment groups: early-abrupt, early-gradual, late-abrupt, and late-gradual. Blood samples were collected from the jugular vein 1 d preweaning, and one day postweaning. Oxylipids concentration was measured by liquid chromatography-tandem MS. Fatty acid profile (including nonesterified fatty acids) was also assessed. Weaning pace, age, pace × age, BW at birth, and sex were included as fixed effect and cohort was included as random effect in the model. Linoleic acid derivatives 13- oxooctadecadienoic (OxoODE) and 9-OxoODE had a greater concentration in calves abruptly weaned when compared with those for gradually weaned calves. Calves weaned gradually showed a greater concentration of 9-hydroxyoctadecadienoic (HODE), 13-HODE, 12,13-dihydroxyoctadecenoic (DiHOME), 9,10-DiHOME, all linoleic acid-derived compared with that for abruptly weaned calves. Anti-inflammatory oxylipid 17,18-dihydroxyeicosatrienoic, an eicosapentaenoic acid derivative, was greater in calves abruptly weaned compared with those gradually weaned. Overall, the pace of weaning affected the plasma concentration of oxylipids, demonstrating that weaning pace affects the oxylipids status involved in inflammation in dairy calves.

## Highlights

• Seventy-one Holstein dairy calves were weaned early or late and abruptly or gradually.
• Abrupt weaning increased anti-inflammatory oxylipid 17,18-DiHETE.
• Linoleic acid-derived proinflammatory oxylipids predominated with gradual weaning.
• Oxylipids are affected by the pace of weaning, regardless of age.

## Graphical Abstract

**Figure:**
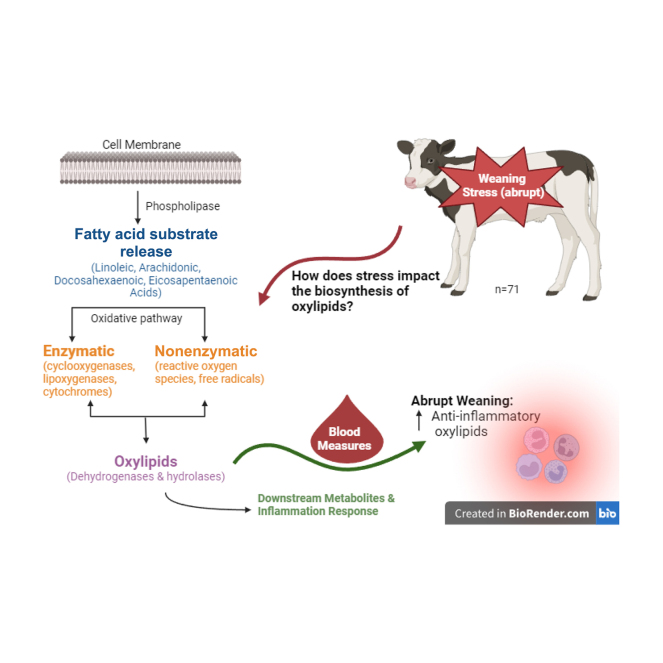
**Summary:** Oxylipids are synthesized from the oxidation of certain polyunsaturated fatty acids by enzymatic or nonenzymatic pathways and are responsible for promoting or resolving inflammation. Their roles are related to the fatty acid source and pathways from which they are derived. However, no evidence in the current literature explains how weaning stress in dairy calves affects the biosynthesis and prevalence of oxylipids. This study aimed to bridge that gap and found that weaning pace can affect the plasma concentration of oxylipids.

Dairy calves are exposed to several stressful events in early life, weaning among them. Depending on how calf weaning is managed, it can affect several growth and health indicators; for example, abrupt weaning decreasing feed intake, ADG, mammary mass, and nonesterified fatty acids (**NEFA**) in blood (Steele et al., 2017). Nonesterified fatty acids are released from triglycerides in the adipose tissues through the enzymes involved in lipolysis, such as hormone-sensitive lipase that has an increased activity and demand during negative energy balance (De Koster et al., 2018). High-blood NEFA concentration has been associated with an elevated risk of developing metabolic issues in dairy cows (McArt et al., 2013). Likewise, high NEFA concentration in vitro also activates oxidative stress and an inflammatory response in calf hepatocytes (Shi et al., 2015). However, because elevated NEFA are usually a result of the increased mobilization of body fat reserves, well fed calves or calves with low body fat may not have this same response (Ockenden et al., 2023).

The mechanisms evaluating inflammation/disease risk and biosynthesis of fatty acids-derived byproducts, also known as oxylipids, have been studied in dairy cows in the past years (Mavangira et al., 2015; Putman et al., 2022). Oxylipids are compounds with potential pro- or anti-inflammatory properties, generated through enzymatic or nonenzymatic oxidation of PUFA. The most common PUFA precursors are proinflammatory n-6 arachidonic (**ArA**) and linoleic acids, and anti-inflammatory n-3 eicosapentaenoic acid (**EPA**) and docosahexaenoic acid (**DHA**). The enzymatic reactions are moderated by cyclooxygenases (**COX**), lipoxygenases (**LOX**), or cytochrome P450 (**CYP**), while the nonenzymatic reactions are triggered by reactive oxygen or nitrogen groups (Mavangira et al., 2015). As with other PUFA, linoleic acid specifically is highly susceptible to oxidation by COX and LOX (Sordillo, 2018). Nonenzymatic reactions triggered by the presence of reactive oxygen and nitrogen groups also can oxidize linoleic acid (Contreras et al., 2020). Regardless of their substrates and biosynthesis pathways, oxylipids involved with inflammation have been identified and their relative amount of pro- to anti-inflammatory activity can be found in the inflammatory profile and response (Putman et al., 2022). Thus, it becomes important to analyze these in the context of different transition time points in a calf's life.

Weaning methods affect NEFA in dairy calves, and NEFA upregulates oxidative stress and inflammation (Li et al., 2022), so determining how weaning pace or age affect the oxylipids profile in dairy calves is essential to better understand the inflammatory mechanisms. Weaning also activates lipolysis and increases the availability of PUFA for oxidation. Previously we have found that abrupt weaning increases NEFA, reflecting peaks in lipolysis rate, and gradual weaning increased BHB, which indicated lower intensity but prolonged lipolysis (Agustinho et al., 2024). However, there are no studies evaluating the effect of stress in dairy calves on the biosynthesis of oxylipids. Therefore, the present study aimed to determine the effect of weaning in dairy calves at different ages and paces on plasma oxylipids.

All animal procedures were approved by the Animal Care and Use Committee at the University of Idaho (#2020–12; #2021–51) and the University of Alberta (#3857). The experiment was conducted on the Dairy Unit at the University of Idaho (Moscow, ID). Experimental design and management were described elsewhere (Wolfe et al., 2023). Briefly, 1-d-old Holstein calves (n = 71, 38.8 ± 4.37 kg, mean ± SD) were blocked by sex and BW at birth (light = <38.5 kg, heavy = >38.5 kg), and then randomly assigned to a 2 × 2 factorial arrangement of treatments using PROC Plate-AN in SAS (version 9.4, SAS Institute Inc., Cary, NC). One factor consisted of weaning age (early vs. late) and the other factor of weaning pace (abrupt vs. gradual), totaling 4 treatments including early-abrupt, early-gradual, late-abrupt, and late-gradual. The study was conducted as 2 cohorts (40 calves in the first cohort and 31 calves in the second cohort).

Calf rearing procedures before weaning are further explained in Wolfe et al. (2023). Briefly, all calves received colostrum replacer within the first hour of arrival at the research unit. Serum total proteins were measured using a refractometer, to ensure an effective transfer of passive immunity. Calves were bottle fed twice a day in equal proportions with milk replacer (Nurture Professional 24–17, Provimi). Milk replacer intake was restricted up to 1,200 g of DM per day for all the treatments, which corresponded to 7.6 L. The weaning transition in the early-abrupt group was conducted from d 39 to d 42 of age; early-gradual from d 35 to d 49 of age; late-abrupt from d 53 to d 56 of age; and late-gradual from d 49 to d 63 of age. The abrupt pace had 4 equal step-downs over 3 d, and the gradual pace had 7 equal step-downs over 14 d. Using this transition method, all the calves weaned at an early age were offered a total of 46.2 kg of milk replacer, and the calves weaned at late age were offered a total of 63.0 kg of milk replacer. Each treatment group had 18 calves, except treatment early-abrupt, which had 17 calves. By allocating 18 animals per treatment, an a priori power analysis, using blood metabolites as outcome variables, estimates that the data analysis can detect a 10% difference between treatments with a 10% CV and 80% power (Berndtson, 1991).

Blood samples were taken from the jugular vein of each calf and collected in Vacutainer tubes containing EDTA to obtain plasma and plain red top tubes to obtain serum. Blood samples were collected on the day before weaning started and 1 d postweaning. Aliquots of 1 mL of plasma samples were stored at −80°C for extraction of oxylipids. Aliquots of 1 mL of serum samples were stored at −20°C for NEFA and fatty acid (**FA**) profile determination.

Oxylipids were extracted from plasma as described by Teixeira et al. (2021) with modifications as described below. Two hundred microliters of plasma was combined with 10 µL of surrogate standard solution, 10 µL of antioxidant solution, and 600 µL of cold methanol. The mix was vortexed for 30 s and centrifuged at 15,000 × *g* for 10 min at 4°C. An Oasis HLB Vacuum 3-mL solid-phase extraction (**SPE**) column (60 mg of sorbent) was coupled to a vacuum manifold (Visiprep SPE, Sigma-Aldrich), and was rinsed with 3 mL of ethyl acetate, followed by 6 mL of methanol, and conditioned with 6 mL of SPE buffer (5% methanol and 0.1% acetic acid in water). The extracted sample was transferred to a disposable borosilicate 5-mL tube mixed with 3.3 mL of water, and later placed in the column. The column was rinsed twice with 6 mL of SPE buffer and dried under vacuum. Samples were dried under nitrogen flow and reconstituted with 50 mL of methanol. The antioxidant solution was similar to that used by Teixeira et al. (2021) except that we used 1.2 µL/mL of EDTA in water. The surrogate standard mix was prepared with 12,13-DiHOME_*d4*, 9-HODE_*d4*, 13-HODE_*d4*, PGE_2__*d9* to a final concentration of 2 µ*M* per compound in methanol. External standards were mixed, and a standard curve was prepared with 6 points ranging from 1.65 to 400 µ*M*.

The oxylipid profile was determined by using a Waters Acquity UPLC coupled to a Waters Xevo TQ-S triple quadrupole MS. The analytes were separated with an InfinityLab Poroshell 120 EC-C18 column and held at 50°C. Mobile phase A consisted of water with 0.1% formic acid and mobile phase B consisted of acetonitrile:methanol (80:20, vol/vol) with 0.1% of formic acid. The oxylipids were detected in a negative electrospray ionization mode. Cone voltages and collision voltages were optimized for each analyte.

The oxylipids relative concentration was found by plotting the ratio of concentration of external standard to concentration of surrogate standard on the x-axis and the ratio of the area of external standard to the area of surrogate standard on the y-axis, as described by Teixeira et al. (2021). Subsequently, the relative concentration was converted to the actual concentration by plotting the ratio of concentration of external standard to concentration of surrogate standard on the x-axis, and the concentration of external standard on the y-axis. In addition, soluble epoxide hydrolase (sEH) product to substrate ratios were calculated to determine the systemic metabolism of PUFA through sEH because Stefanovski et al. (2020) demonstrated that epoxide-diol ratios from sEH correlated strongly with each other.

The serum concentration of NEFA was determined by using in vitro enzymatic colorimetric assays (Wako series NEFA–HR (2); Fujifilm Wako Chemicals), according to manufacturer instructions. Lipids in serum samples were extracted by chloroform:methanol (2:1) and fractionated using Sep-Pak aminopropyl cartridges as previously described (Tsai et al., 2017). Methylated NEFA samples were then processed via GC. Gas chromatography requires methylated samples to improve volatility, stability, and detectability. As done with Tsai et al. (2017), derivatized samples were needed to make them sufficiently volatile to be eluted at reasonable temperatures without thermal decomposition.

The FA profiles of methylated lipid samples were determined using an Agilent 7890A GC system (Agilent Technologies) as previously reported (Tsai et al., 2017). Fatty acids are presented as the method utilized performs quantitative analysis by the percentage peak area but with compensation for relative component sensitivity. This method may have limitations for quantifying the PUFA substrate availability because it was very low.

All statistical analyses were conducted using the MIXED procedure of SAS (version 9.4, SAS Institute Inc., Cary, NC). Weaning pace, age, pace × age, BW at birth, and sex were included as fixed effect and cohort was included as random effect in the model. Analytes and NEFA concentration from preweaning samples were used as covariate in the model of each respective compound. Interactions were investigated using the “slice” option. Differences were declared significant at *P* ≤ 0.05.

Weaning pace can affect calves' NEFA concentration and possibly their energy balance accordingly. There was an increase in NEFA concentration in calves weaned abruptly (*P* < 0.01; Table 1), regardless of the weaning age. Abruptly weaned animals may have a more evident negative energy balance (**NEB**) that may have been a consequence of less time in abrupt pace (Wolfe et al., 2023). The NEFA are released from triacylglycerol in adipose tissue by hormone-sensitive lipase and other lipases. (De Koster et al., 2018). Our previous study found that although abruptly weaned calves have a higher NEFA (and later NEB) peak postweaning, gradually weaned calves may have prolonged NEB because of the FA partially oxidized to ketone bodies in the liver, reflecting higher BHB concentration in plasma (Agustinho et al., 2024). Previous studies found that calves weaned abruptly had greater NEFA concentrations and had a reduced ME intake and BW (Steele et al., 2017) than those weaned gradually. Because oxylipid biosynthesis is dependent on PUFA substrate availability as well as enzymatic activity (Contreras et al., 2020), we analyzed the FA profiles to inform how proinflammatory and anti-inflammatory oxylipids change.

**Table 1:** Nonesterified fatty acids (NEFA) in serum and oxylipid concentration and ratio in plasma of Holstein dairy calves weaned at different ages and paces

Item^1^	Age		Pace		SEM	*P*-value		
	Early	Late	Abrupt	Gradual		Age	Pace	Age × pace
NEFA, mEq/L	0.19	0.18	0.261	0.11	0.034	0.76	<0.01	0.07
Analyte, μ*M*								
11,12-EET	0.172	0.044	0.173	0.043	0.055	0.11	0.10	0.40
9,10-DiHOME	0.535	0.769	0.361	0.943	0.188	0.02	<0.01	0.12
12,13-DiHOME	6.966	8.490	5.934	9.522	1.589	0.07	<0.01	0.31
17,18-DiHETE	1.587	1.248	1.769	1.065	0.318	0.11	<0.01	0.08
11,12-DHET	0.279	0.173	0.293	0.160	0.045	0.10	0.04	0.28
13-OxoODE	12.101	11.515	16.779	6.837	2.257	0.85	<0.01	0.42
9-OxoODE	8.021	5.830	9.366	4.485	1.556	0.32	0.03	0.89
12,13-EpOME	20.24	20.98	22.58	18.63	3.81	0.88	0.43	0.17
9,10-EpOME	10.79	9.90	12.89	7.79	2.03	0.75	0.07	0.54
13-HODE	22.84	21.39	21.30	22.92	2.02	0.61	0.57	0.97
9-HODE	12.41	12.05	12.37	12.09	1.36	0.85	0.88	0.97
11-HETE	0.251	0.227	0.249	0.229	0.039	0.67	0.72	0.09
12-HETE	1.094	1.025	1.174	0.945	0.163	0.77	0.31	0.73
Ratio, C/C								
9-HODE/9-OxoODE	3.19	3.42	2.90	3.71	0.707	0.80	0.38	0.22
13-HODE/13-OxoODE	5.78	4.44	2.39	7.82	1.589	0.54	0.02	0.39
9,10-EpOME/9,10-DiHOME	29.19	21.43	38.42	12.19	5.436	0.28	<0.01	0.70
12,13-EpOME/12,13-DiHOME	3.71	3.25	5.18	1.78	0.810	0.68	<0.01	0.81

1 NEFA = nonesterified fatty acids; EET = epoxyeicosatrienoic acid; DiHOME = dihydroxyoctadecenoic acid; DiHETE = dihydroxyeicosatrienoic acid; OxoODE = oxooctadecadienoic acid; EpOME = epoxyoctadecenoic acid; HODE = hydroxyoctadecadienoic acid; HETE = hydroxyeicosatetraenoic acid; C/C = consume/create ratio.

On specific FA percentages (Table 2), this study found that a later weaning age increased C19:0 (nonadecanoic acid; *P* = 0.03) and C20:5 n-3 (EPA; *P* = 0.02). These and other FA were unaffected by weaning pace. These results indicate how age and pace of weaning can affect FA profile differently and independent of oxylipid biosynthesis.

**Table 2:** Fatty acid percentage in plasma of Holstein dairy calves with different weaning strategies

Fatty acid,^1^ %	Age		Pace		SEM	*P*-value		
	Early	Late	Abrupt	Gradual		Age	Pace	Age × pace
C14:1	1.495	1.710	0.530	2.675	1.593	0.86	0.08	0.70
C16:0	22.18	17.89	22.69	17.39	2.199	0.12	0.05	0.98
C17:0	2.222	2.807	1.161	3.869	1.290	0.71	0.08	0.38
C18:2 n-6	0.187	1.237	1.215	0.210	0.864	0.38	0.40	0.36
C20:0	0.334	0.399	0.144	0.589	0.320	0.89	0.32	0.19
C19:0	0.350	1.330	0.834	0.846	0.503	0.03	0.98	0.95
C20:5 n-3	0.031	0.740	0.309	0.462	0.206	0.02	0.50	0.91
C24:1	0.669	0.372	0.169	0.872	0.436	0.44	0.07	0.84
C22:5 n-6	0.0050	0.0403	0.0439	0.0014	0.0321	0.43	0.34	0.34
C22:5 n-3	0.766	0.332	0.468	0.630	0.348	0.27	0.67	0.71

1 C18:2 = linoleic acid, C20:0 = arachidic acid, C20:5 = eicosapentaenoic acid.

Independent of the treatment, the most abundant oxylipids observed in dairy calves were 13-HODE, 12,13-EpOME, 13-oxoODE, 9-HODE, and 12,13-DiHOME. Although these oxylipids are biosynthesized through different pathways, they share n-6 PUFA, linoleic acid, as their FA precursor. Linoleic acid is one of the predominant FA in calves (Śpitalniak-Bajerska et al., 2020). Overall, linoleic-acid-derived oxylipids are vital in determining potential inflammatory responses.

The most abundant oxylipids in dairy cows were shown to be 9-HODE and 13-HODE (Putman et al., 2022), both of which are derivatives of linoleic acid, with 9-HODE being proinflammatory and 13-HODE being pro- and anti-inflammatory. Mavangira et al. (2015) found that 13-HODE concentrations, related to lipolytic activity, increase in periparturient and mastitic dairy cows, whereas our study showed no effect of weaning methods on the 13-HODE in dairy calves. 13-Hydroxyoctadecadienoic can initiate macrophage anti-inflammatory polarization during lipolysis and acts as a peroxisome proliferator-activated receptor (**PPAR**)-γ ligand that promotes adipogenesis and lipogenesis (Lee et al., 2016). There was no effect of the weaning age or pace on the oxylipids 9-HODE and 13-HODE (*P* > 0.50). Although the previous studies may have observed changes in 9-HODE and 13-HODE abundance, there were none observed in the current study's calves, even if these oxylipids are the most abundant in adult ruminants.

The linoleic acid derivatives, anti-inflammatory ketones 9-OxoODE and 13-OxoODE showed a greater concentration in calves weaned abruptly rather than gradually (*P* = 0.03 and *P* < 0.01 respectively; Table 1); which may demonstrate that early weaning levels of HODE are rapidly metabolized to oxoODE to initiate the resolution phase of inflammation. An endogenous ligand to PPAR-γ, 9-oxoODE downregulates inflammatory responses in macrophages and other innate immune cells (Altmann et al., 2007). 13-Oxooctadecadienoic is a potent ligand for an anti-inflammatory nuclear receptor and reduces proinflammatory IL-8 through PPAR-γ activation in human colonic epithelial cells (Altmann et al., 2007). Currently, abrupt weaning may induce a peak in the production of HODE that are metabolized to oxoODE by the time of sample collection. In contrast, the gradually weaned calves may exhibit a low rate or extended period of lipolysis that leads to a constant production of HODE, and this was reflected in the higher concentration of these oxylipids at the time of sample collection. Greater concentration of anti-inflammatory oxylipids indicates that animals weaned abruptly were possibly starting to resolve their inflammatory responses shortly after weaning, meaning that weaning can be a rapid enough change to possibly induce very short-lived changes in inflammatory oxylipids that are beyond the samples collected for this study. Further analysis of oxylipids involved in inflammatory processes could elucidate these findings.

The proinflammatory epoxide 9,10-epoxyoctadecenoic acid (EpOME; linoleic acid derived) was not affected by age or pace of weaning (*P* = 0.75 and 0.09, respectively). However, the opposite was observed regarding the proinflammatory diol 9,10-DiHOME acid (*P* < 0.01), where calves weaned gradually had a greater concentration; 9,10-DiHOME was also greater in calves at late age (*P* = 0.02). Although the 12,13-EpOME were not affected by weaning pace or age (*P* = 0.17), 12,13-DiHOME had a greater concentration in calves weaned gradually (*P* < 0.01). Previous studies note the downstream metabolites of EpOME (DiHOME) are far more potent than their parent oxylipid due to their leukotoxin diols (Moghaddam et al., 1997). Likewise, DiHOME is produced during an oxidative burst, which is a critical component of the innate immune defense against pathogens (Mosca et al., 2020). Our findings of gradual weaning having increased DiHOME demonstrate how these treatments have more oxidative, inflammatory response and thus greater levels of a more potent proinflammatory oxylipid.

When analyzing epoxide-diol ratios between oxylipids to better assess the balance between pro- and anti-inflammatory actions we found there was no detectable effect of age (*P* = 0.80) or pace (*P* = 0.38) on the ratio of 9-HODE to 9-OxoODE. However, although the ratio of 13-HODE to 13-OxoODE increased with a gradual weaning transition (*P* = 0.02), and the ratios of 9,10-EpOME/9,10-DiHOME (*P* < 0.01), and 12,13-EpOME/12,13-DiHOME (*P* < 0.01) decreased with a gradual weaning, all these oxylipids are linoleic-derived. As demonstrated by Stefanovski et al. (2020), these epoxide-diol ratios are principally metabolized by sEH and are strongly correlated; however, further studies should also analyze sEH activity through analysis of multiple indices to prevent conflicting interpretation. Thus, it must be noted that use of only 4 ratios to infer sEH activity could be another limitation of the present study. The notable difference among these oxylipids is that they are generated through LOX (9-HODE/9-OxoODE) and COX (13-HODE/13-OxoODE) or CYP (9,10-EpOME/9,10-DiHOME and 12,13-EpOME/12,13-DiHOME). This implies that lipid compounds metabolized from LOX and COX can increase, while those metabolized with cytochromes decrease, with gradual weaning.

The epoxide 11,12-epoxyeicosatrienoic acid (**EET**), derived from ArA through the enzyme CYP and diol 11,12-dihydroxyeicosatrienoic acid (**DHET**), posteriorly synthetized from 11,12-EET by sEH followed a similar pattern to the above EpOME and DiHOME. It was found that 11,12-DHET presented a greater concentration in calves weaned at an abrupt pace (*P* = 0.04, Table 1). Mavangira et al. (2021) found that the ratio in mastitic cows was greater for the 11,12-EET/11,12-DHET pair compared with that of the healthy control cows. The significance of the further metabolism of EET by sEH is that many of the beneficial anti-inflammatory functions of the initial epoxygenase metabolites are lost (Sordillo, 2018), which is interesting because the current study shows increases in 11,12-DHET when calves are weaned abruptly that are similar to that of cows undergoing a mastitis challenge.

We also analyzed oxylipids involved with anti-inflammatory actions derived from n-3 PUFA, EPA, and DHA. The oxylipid 17,18-DiHETE, derived from EPA, was not significantly affected by age (*P* = 0.11) but was increased in the calves weaned abruptly (*P* < 0.01; Table 1). Previous studies found that 17,18-DiHETE has anti-inflammatory actions (Putman et al., 2022) and is the diol metabolite of the anti-inflammatory 17,18-epoxyeicosatetraenoic acid (Morin et al., 2010). The increased oxylipids concentration may therefore be because of anti-inflammatory compounds being produced in response to inflammation.

In summary, this study investigated how weaning methods affect plasma oxylipids and serum NEFA profile in dairy calves. We found that linoleic acid anti-inflammatory oxylipids 13-OxoODE and 9-OxoODE were greater in calves weaned abruptly. Calves weaned gradually showed a greater concentration of proinflammatory 9-HODE, 12,13-DiHOME, 9,10-DiHOME, and anti-inflammatory13-HODE. Our findings demonstrate how gradual weaning has more increases in proinflammatory oxylipids compared with abrupt weaning. These results indicate how oxylipids are affected by the pace of weaning but not the age at weaning.
